# GRP78 confers the resistance to 5-FU by activating the c-Src/LSF/TS Axis in hepatocellular carcinoma

**DOI:** 10.18632/oncotarget.5603

**Published:** 2015-09-10

**Authors:** Yan-jiao Gu, Hong-dan Li, Liang Zhao, Song Zhao, Wu-bin He, Li Rui, Chang Su, Hua-chuan Zheng, Rong-jian Su

**Affiliations:** ^1^ Pathology Department, The First Affiliated Hospital of Liaoning Medical College, Jinzhou, China; ^2^ Central Laboratory, Liaoning Medical College, Jinzhou, China; ^3^ Pharmacy Department, Liaoning Medical College, Jinzhou, China; ^4^ Veterinary Medicine Department, Liaoning Medical College, Jinzhou, China; ^5^ Cancer Research Center, Key Laboratory of Brain and Spinal Cord Injury of Liaoning Province and Laboratory Animal Center, The First Affiliated Hospital of Liaoning Medical College, Jinzhou, China

**Keywords:** GRP78, chemoresistance, 5-FU, LSF, TS

## Abstract

5-FU is a common first-line chemotherapeutic drug for the treatment of hepatocellular carcinoma. However the development of acquired resistance to 5-FU confines its clinical usages. Although this phenomenon has been the subject of intense investigation, the exact mechanism of acquired resistance to 5-FU remains elusive. Here, we report that over-expression of GRP78 contributes to acquired resistance to 5-FU in HCC by up-regulating the c-Src/LSF/TS axis. Moreover, we found that the resistance to 5-FU conferred by GRP78 is mediated by its ATPase domain. The ATPase domain differentially increased the expression of LSF, TS and promoted the phosphorylation of ERK and Akt. We further identified that GRP78 interacts physically with c-Src through its ATPase domain and promotes the phosphorylation of c-Src, which in turn increases the expression of LSF in the nucleus. Together, GRP78 confers the resistance to 5-FU by up-regulating the c-Src/LSF/TS axis via its ATPase domain.

## INTRODUCTION

Hepatocellular carcinoma (HCC) is the fifth common cancer worldwide whose mortality rate parallels with its incidence [1]. Rapid growth and early vascular invasion are the most important characteristics of HCC [2-4]. The treatment options for HCC depend on the stages and grades of this disease. For the patients with advanced stages, systemic therapy is still a very important option. To date, 5-FU is still a common therapeutic drug for the systemic therapy of hepatocellular carcinoma. For example, a combination of cisplatin, IFN, doxorubicin and 5-FU (PIAF) was being widely used for the treatment of advanced HCC [5-7]. Unfortunately, it has improved survival in a very limited extent [8].

The development of 5-FU resistance is a common phenomenon in HCC patients who underwent 5-FU treatment and confines its use in clinical practice [9-11]. Many signaling pathways have been demonstrated to contribute to the resistance to 5-FU [12, 13]. Among them, the LSF/TS axis plays a decisive role in the development of 5-FU resistance [14]. Late SV40 factor (LSF) is overexpressed in HCC and functions as a transcriptional activator and repressor, activating the transcription of target genes including SAA3, IL-4, α-globin and PAX6[15]. Recent progress has identified TS, which encodes the rate limiting enzyme in the synthesis of dTTP, as one of the downstream regulators of LSF [14]. LSF contributes largely to the development of 5-FU resistance by binding to TS promotes, up-regulating the expression of TS.

Glucose-regulated protein 78 kDa (GRP78) is a member of the heat shock protein 70 family [16, 17] and protects cells from apoptosis under stress conditions [18, 19]. It is overexpressed in many human cancers and plays critical role in the regulation of cellular proliferation [20, 21], invasion [22], metastasis and survival [23, 24]. Many data have demonstrated that overexpression of GRP78 causes the resistance to many chemotherapeutic agents including cisplatin, (−)-epigallocatechin gallate, temozolomide, doxorubicin and 5-FU in many human solid tumors such as breast [25, 26], lung, gastric cancers [27-29]. Up to now, little is known about the role of GRP78 in the development of 5-FU resistance in HCC.

In this report, we investigated the role of 5-FU in regulation of hepatocellular carcinoma sensitivity to 5-FU and the underlying mechanism by which GRP78 confers resistance to 5-FU. We found that high GRP78 level enhanced resistance to 5-FU. Overexpression of GRP78 activated c-Src phosphorylation which, in turn, elevated LSF/TS axis in HCC cells. Thus these results provide new insights into the mechanism of 5-FU resistance, and treatment of HCC with 5-FU in combination with PP2 increased the efficacy of 5-FU.

## RESULTS

### Over-expression of GRP78 confers resistance to 5-FU in HCC cells *in vitro*

**Figure 1:**
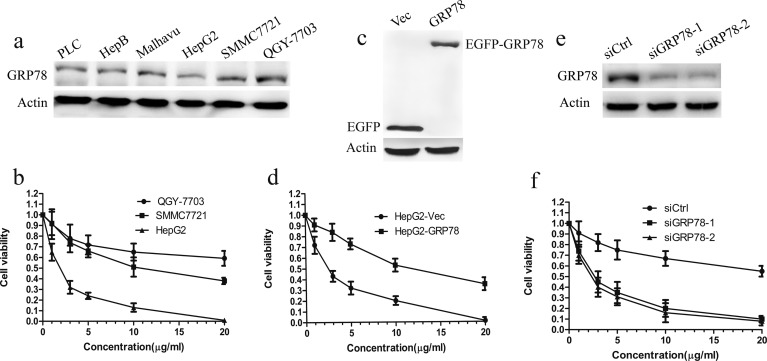
Overexpression of GRP78 confers the resistance to 5-FU in HCC cells **a.** Western blot analysis of GRP78 in a panel of 6 HCC cell lines, including QGY-7703, SMMC7721, Malhavu, PLC, Hep3B and HepG2. **b.** Cell viability analysis of the sensitivity of QGY-7703, SMMC7721 and HepG2 cells to 5-FU, Data are representative of three independent experiments (mean±S.D.). **c.** Western blot analysis of EGFP-GRP78 in HepG2 cells. HepG2 cells were transfected with EGFP tagged wild type GRP78 for 48h and analyzed for western blot analysis using anti-GFP antibody. **d.** Cell viability analysis of the sensitivity of HepG2-GRP78 cells to 5-FU. Data are representative of three independent experiments (mean±S.D.). **e.-f.** Two independent siRNAs targeting GRP78 enhance response to 5-FU. **e.** QGY-7703 cells were transfected with siRNAs against GRP78 for 48 h and analyzed for western blot analysis using anti-GRP78 antibody. **f.** Cell viability analysis of the sensitivity of QGY-7703 cells transfected to siRNAs against GRP78 to 5-FU. Data are representative of three independent experiments (mean±S.D.).

Over-expression of GRP78 is associated with resistance to chemical drugs in many human cancers. However, the contribution of GRP78 to the resistance to 5-FU in HCC has not been previously explored. Using western blot, we examined GRP78 levels in a panel of 6 HCC cell lines and found a high variation in expression. As shown in Figure 1a, QGY-7703 cells expressed GRP78 at higher level as compared with other cell lines. In contrast, HepG2, PLC and Hep3B cells expressed GRP78 at relatively lower level. Malhavu, SMMC7721 cells expressed GRP78 at intermediate level. Subsequent cell viability analysis in QGY-7703, SMMC7721 and HepG2 cells showed the sensitivity to 5-FU differs widely in these HCC cell lines. The response efficacy to 5-FU is the lowest in QGY-7703 cells, by contrast it is the highest in HepG2 cells, raising the possibility that the expression level of GRP78 may be associated with the sensitivity of HCC cells to 5-FU (Figure 1b).

To investigate whether GRP78 overexpression is sufficient to confer the resistance to 5-FU, HepG2 cells were engineered to stably express EGFP (HepG2-Vec) or EGFP-tagged GRP78 (HepG2-GRP78) (Figure 1c). HepG2 cells were chosen because they expressed GRP78 at a relatively lower level as compared with the other cells we have examined. When comparing the sensitivity to 5-FU, overexpression of GRP78 significantly increased cell viability in HepG2 cells, reaching an IC50 that was almost some 5 fold higher than that of HepG2-Vec cells (Figure 1d). By contrast, down-regulation of GRP78 in QGY-7703 cells using GRP78 specific siRNAs markedly decreased cell viability when treated with 5-FU, indicating that GRP78 could confer resistance to 5-FU in HCC (Figure 1e, 1f).

### The ATPase domain of GRP78 determines the sensitivity to 5-FU in HCC cells

**Figure 2:**
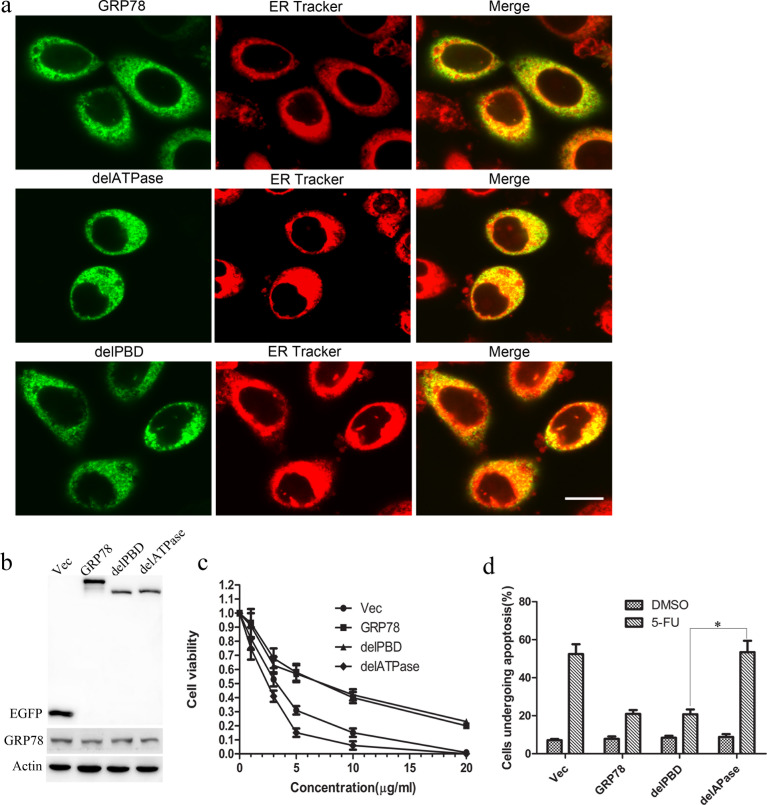
GRP78 confers the resistance to 5-FU by its ATPase domain **a.** Cellular localization of delATPase and delPBD in HepG2 cells. HepG2 cells transfected with delATPase or delPBD domain were stained with TRITC-conjugated ER tracker and observed using confocal microscope (Scale bar: 25 μM). **b.** Western blot analysis of the expression of EGFP tagged GRP78, delPBD and delATPase in HepG2 cells using anti-GFP antibody after 48h of transfection. **c.** Cell viability analysis of the sensitivity of HepG2 cells overexpressing EGFP tagged wild type GRP78, delPBD or delATPase to 5-FU. Data are representative of three independent experiments (mean±S.D.). **d.** Schematic show of flow cytometry analysis of cell apoptosis in HepG2 cells overexpressing EGFP tagged GRP78, delPBD or delATPase when treated with 5-FU at a concentration of 10 μg/ml. Data are representative of three independent experiments (mean±S.D.). *P < 0.05 as determined by two-way ANOVA.

To determine the role of ATPase and PBD domains of GRP78 in the acquired resistance to 5-FU conferred by GRP78, we established EGFP tagged ATPase or PBD domain deleted mutant (abbreviated as delATPase and delPBD) (Figure S1a, b) and introduced these mutants into HepG2 cells using lipofectamine2000. As indicated by TRITC-conjugated ER-tracker, delATPase and delPBD mutants are localized in the ER and cytoplasm, suggesting that deletion of ATPase and PBD did not alter the cellular localization of GRP78 (Figure 2a). Western blot analysis showed that GRP78, delATPase or delPBD was overexpressed in HepG2 cells as compared with endogenous GRP78 (Figure 2b).

We next determined the function of ATPase and PBD domain in GRP78 mediated 5-FU resistance in HepG2 cells using cell viability assay. As shown in Figure 2c, overexpression of delPBD mutant of GRP78 in HepG2 cells shared a parallel cell viability ratio as compared with that of GRP78. By contrast, overexpression of delATPase mutant caused a marked decrease in cell viability ratio as compared with that of GRP78, indicating that GRP78 determines the sensitivity of HCC cells to 5-FU by its ATPase domain. This conclusion was further supported by the fact that overexpression of GRP78 or its delPBD mutant caused a marked decrease in 5-FU induced cell apoptosis as compared with that in cells overexpressing delATPase (Figure 2d, S1c). In addition, we found that overexpression of delPBD promoted the invasion and migration of HepG2 cells. However, overexpression of delATPase inhibited the invasion and migration of HepG2 cells, suggesting that GRP78 promotes the invasion and migration of HepG2 cells by its ATPase domain (Figure S2).

### LSF is essential for GRP78 mediated resistance to 5-FU

**Figure 3:**
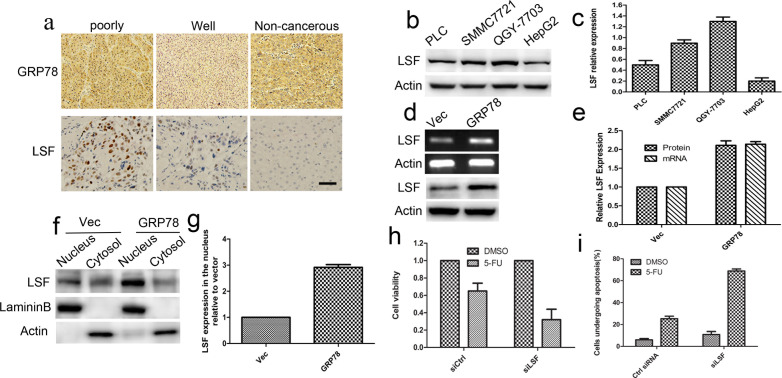
LSF is essential for GRP78 mediated resistance to 5-FU **a.** Immunohistochemical staining of the expression of GRP78 and LSF in 44 cases of surgery resected tissue samples. Non-cancerous tissues were stained as control (Scale bar: 50 μM). **b.-c.** Western blot analysis of LSF in QGY-7703, SMMC7721 and HepG2 cells. **d.-e.** Overexpression of GRP78 increased LSF in transcriptional level. **d.**, Quantitative RT-PCR and western blot analysis of LSF in HepG2 cells overexpressing EGFP-GRP78. **e.**, Schematic show of LSF expression at mRNA and protein levels in HepG2 cells overexpressing EGFP-GRP78. Data were presented with mean±S.D. **f.-g.** Western blot analysis of LSF in the cytosol and nuclear extracts in HepG2 cells overexpressing EGFP-GRP78. **h.** Cell viability analysis of the sensitivity of HepG2-GRP78 cells transfected with LSF specific siRNA to 5-FU. Data are representative of three independent experiments and were presented as mean±S.D. **i.** Schematic show of flow cytometry analysis of cell apoptosis in LSF knockdown HepG2-GRP78 cells when treated with 5-FU at a concentration of 10 μg/ml. Data are representative of three independent experiments and were presented as mean±S.D.

To identify whether LSF is one of GRP78-downstream genes, we investigated whether GRP78 could increase LSF expression. We first examined the expression of GRP78 and LSF in 44 cases of surgery resected HCC tissue sample using immunohistological staining. The clinicopathological data were summarized in Table 1. As shown in Figure 3a, both GRP78 and LSF were expressed in HCC tissue samples. GRP78 was expressed in the cytoplasm, while LSF was detected predominantly in the nucleus. Quantitative analysis showed that both GRP78 and LSF were expressed at higher levels in poorly differentiated tissue samples as compared with that in well differentiated tissue samples (Tables 2, 3). Statistical spearman analysis indicated that the expression level of Grp78 was positively correlated with that of LSF in HCC (Table 4) (*p*<0.05). These findings were extended by western blot analysis of LSF expression in hepatocellular carcinoma cell lines including QGY-7703, SMMC7721, PLC and HepG2. As expected, QGY-7703 cells expressed LSF at considerably higher level as compared with HepG2, and SMMC7721, while HepG2 cells expressed LSF at relatively lower level (Figure 3b-3c).

**Table 1:** The clinocopathological data of 44 HCC tissue samples

Clinicopathologic parameters	Number of cases
**Age (years)**	
<60	31
≥60	13
**Gender**	
Male	33
Female	11
**HBsAg**	
No	18
Yes	26
**Degree of differentiation**	
well and moderately	23
poorly	21
**Portal invasion**	
No	23
Yes	21

**Table 2:** GRP78 expression in HCC tissue samples

Grade	GRP78				
	1	2	3	r	*p*
well	13	6	4	*0.869*	*0.000*
poorly	3	5	13		
total	16	11	17		

**Table 3:** LSF expression in HCC tissue samples

grade	LSF				
	1	2	3	r	*p*
well	13	8	2	*0.840*	*0.000*
poorly	4	5	12		
total	17	13	14		

**Table 4:** The correlation of GRP78 and LSF in HCC tissue samples

GRP78						
LSF	Score	1	2	3	r	*p*
	1	11	5	1	*0.946*	*0.000*
	2	4	4	5		
	3	1	2	11		

We next investigated whether overexpression of GRP78 elevated LSF expression in HepG2 cells. Using RT-PCR, we found a marked increase of LSF mRNA in HepG2-GRP78 cells as compared with that in HepG2-Vec cells (Figure 3d, upper). Similar results were obtained using western blot analysis (Figure 3d, bottom). Further quantitative analysis revealed that LSF mRNA increased at a parallel extent with that of LSF protein in HepG2-GRP78 cells, suggesting that GRP78 regulates LSF expression at the transcriptional level (Figure 3e). The effect of GRP78 overexpression on the nuclear expression of LSF was also examined by fractionating the cytosolic and nuclear compartments and analyzing LSF expression using Western blot. We found that LSF was expressed at higher level in the nucleus in HepG2-GRP78 cells than that in HepG2-Vec cells (Figure 3f, 3g). This conclusion was also confirmed in HepG2-GRP78 and HepG2-Vec cells by immunocytochemical staining using anti-LSF antibody (Figure S3a). Our results further revealed that knockdown of GRP78 in QGY-7703 cells significantly decreased LSF (Figure S3b). These data suggested that overexpression of GRP78 could increase LSF level transcriptionally, indicating LSF lies downstream of GRP78 in HCC.

We next determined LSF is involved in the GRP78 mediated resistance to 5-FU. HepG2-GRP78 cells were transfected with siRNA against LSF (Figure S3c) and the content of growth inhibition was examined using cell viability assay in the presence of 5-FU. We found that Knockdown of LSF significantly decreased cell viability of HepG2-GRP78 cells when treated with 5-FU (Figure 3h). Flow cytometry revealed that knockdown of LSF enhanced cell apoptosis induced by 5-FU (Figure 3i, S3d). These data suggested that knockdown of LSF could revert the resistance to 5-FU conferred by GRP78, indicating the critical role of LSF in GRP78 mediated resistance to 5-FU in HCC.

### LSF/TS axis is involved in GRP78 mediated 5-FU resistance in HCC

**Figure 4:**
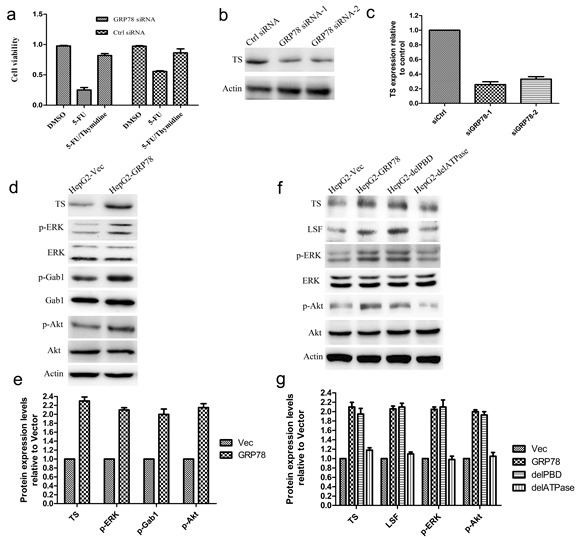
The LSF/TS axis is involved in GRP78 mediated 5-FU resistance in HCC **a.** Exogenous thymidine inhibited cell death induced by 5-FU. QGY-7703 cells transfected with GRP78 specific siRNAs were pretreated by addition of exogenous thymidine and the sensitivity to 5-FU was examined by cell viability assay. **b.-c.** Western blot analysis of TS in QGY-7703 cells transfected with siRNAs against GRP78. **d.-e.** Western blot analysis of TS, p-ERK, p-Akt and p-Gab1 in HepG2 cells overexpressing EGFP-GRP78. **f.-g.** Western blot analysis of TS, LSF p-ERK, p-Akt in HepG2 cells overexpressing EGFP tagged GRP78, delPBD or delATPase.

Thymidylate synthase (TS) has been identified as one of LSF downstream genes that determine the sensitivity to 5-FU. To determine whether the LSF/TS axis is involved in GRP78 conferred 5-FU resistance, We treated QGY-7703 cells with exogenous thymidine (20 μM) in the presence of 5-FU and observed the enhanced cell death induced by 5-FU in QGY-7703 cells transfected with GRP78 specific siRNAs could be rescued by addition of exogenous thymidine, indicating that the resistance to 5-FU conferred by GRP78 is dependent on TS (Figure 4a).

We next down-regulated GRP78 level using siRNA in QGY-7703 cells and found that knockdown of GRP78 caused a significant decrease in the expression of TS (Figure 4b, 4c). As a corollary, overexpression of GRP78 in HepG2 cells increased TS expression (Figure 4d, 4e), indicating that the LSF/TS axis is involved in GRP78 conferred resistance to 5-FU. We also found that overexpression of GRP78 significantly increased the phosphorylation of ERK, Gab1 and Akt, but did not affected the expression of these proteins (Figure 4d, 4e).

To further identify the role of ATPase or PBD domain of GRP78 in the regulation of the LSF/TS axis, LSF and TS levels in HepG2 cells overexpressing delATPase or delPBD were determined using western blot. As compared with GRP78, overexpression of delATPase caused a marked decrease in TS and LSF, while delPBD did not affect the expression of TS and LSF, indicating that GRP78 up-regulated the LSF/TS axis via its ATPase domain (Figure 4f, 4g). As indicated by Figure 4f and 4g, the ATPase domain of GRP78 also plays critical roles in phosphorylation of ERK and Akt.

### GRP78 interacted physically with c-Src and promoted the phosphorylation of c-Src

**Figure 5:**
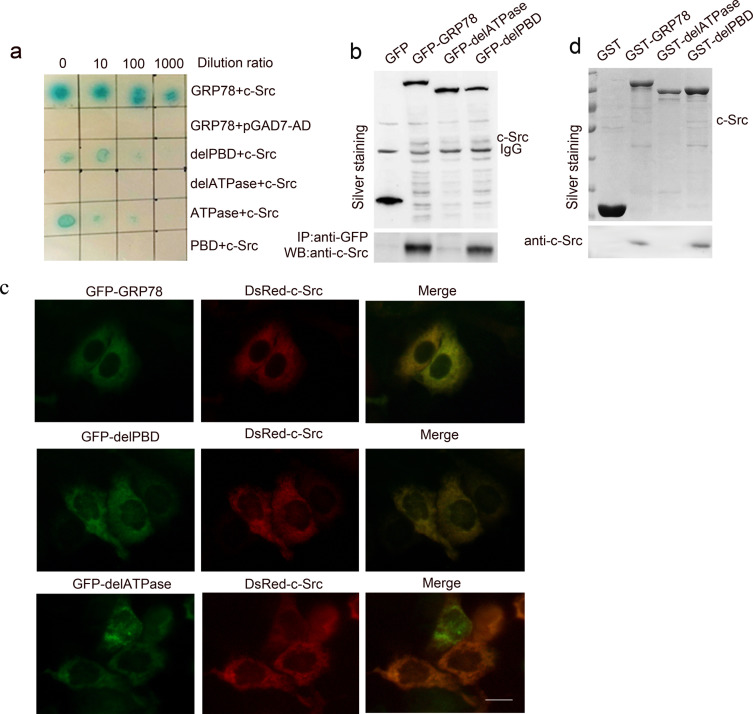
GRP78 interacted physically with c-Src **a.** Yeast two hybrid screening of GRP78 interacting proteins in a fetal liver cDNA library using GRP78, delATPase, delPBD, ATPase and PBD as the baits. **b.** Anti-EGFP co-immunoprecipitation identified the interaction between GRP78 and c-Src. HepG2 cells overexpressing EGFP tagged GRP78 or delPBD or delATPase were lysed using 1% NP-40 buffer and the lysates were precipitated with anti-EGFP. The precipitations were subjected to Western blot using anti-c-Src antibody. **c.** Inverted fluorescent microscope observation of the co-localization of c-Src with GRP78 or delPBD in HepG2 cells co-transfected with dsRed-tagged c-Src and EGFP-tagged GRP78, delPBD or delATPase (Scale bar: 25 μM). **d.** GST-pulldown analysis demonstrated the direct interaction between GRP78 and c-Src. HepG2 cells were lysed using 1% NP-40 buffer and the lysates were precipitated with GST tagged GRP78,delPBD or delATPase. The precipitations were subjected to Western blot using anti-c-Src antibody.

To identify the signaling molecules responsible for activating the LSF/TS axis, two-hybrid (Y2H) screening was performed using human GRP78 as the bait in human Fetal Liver cDNA Library. We identified c-Src as a potential GRP78 interacting protein. GRP78-c-Src interaction was determined by colony formation on yeast SD-Leu-Trp-His-Aba (SD-4) selection media and plate assays for β-galactosidase activity (Figure 5a line 1-2). On this basis, we explored the role of ATPase and PBD domain in the interaction between GRP78 and c-Src by Y2H and found that this interaction was mediated by the ATPase but not PBD domain (Figure 5a line 3-6).

We next performed anti-EGFP co-immunoprecipitation in HepG2 cells transfected with EGFP-tagged GRP78, delATP and delPBD. Anti-EGFP co-immunoprecipitation showed that GRP78 interacted with c-Src through the ATPase domain (Figure 5b). This conclusion was further supported by co-localization of GRP78 or delPBD with c-Src in HepG2 cells co-transfected with DsRed tagged c-Src and EGFP-tagged GRP78 or delPBD; however no co-localization is observed in HepG2 cells co-transfected with c-Src and EGFP tagged delATPase (Figure 5c).

To address whether the interaction between GRP78 and c-Src is direct, we further performed GST-pulldown in HepG2 cells using GST-tagged GRP78, delPBD or delATPase as baits. As shown in Figure 5d, c-Src was presented in the precipitations of GST-GRP78 and delPBD, but not presented in GST-delATPase precipitation. These data suggested that GRP78 could interact directly with c-Src by its ATPase domain.

**Figure 6:**
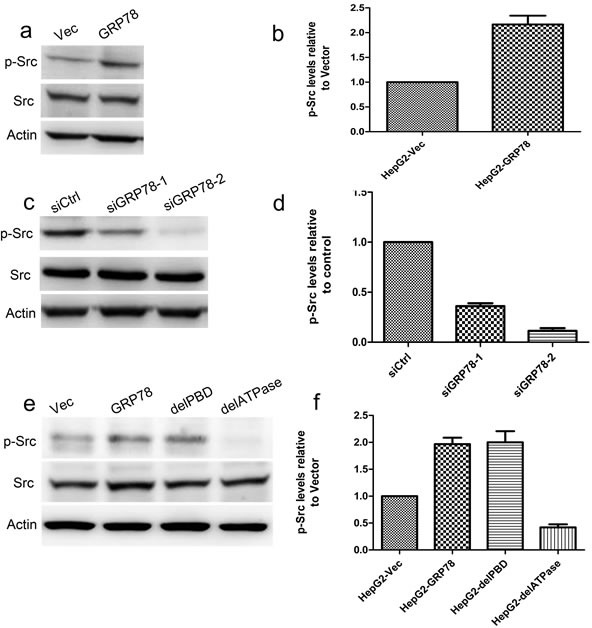
GRP78 promotes the phosphorylation of c-Src via its ATPase domain **a.-b.** Western blot analysis of the phosphorylation of c-Src in HepG2 cells overexpressing EGFP-tagged GRP78 **c.-d.** Western blot analysis of the phosphorylation of c-Src in QGY-7703 cells transfected with siRNAs against GRP78. **e.-f.** Western blot analysis of the phosphorylation of c-Src in HepG2 cells overexpressing EGFP tagged wild type GRP78, delPBD or delATPase.

Since GRP78 could directly interact with c-Src through its ATPase domain. We further investigated whether GRP78 promotes c-Src phosphorylation. Western blot analysis showed that overexpression of GRP78 in HepG2 cells caused a significant increase in the phosphorylation of c-Src (pY416) (Figure 6a-6b), while knockdown of GRP78 in QGY-7703 cells significantly decreased c-Src phosphorylation (Figure 6c-6d). We further examined the role of ATPase and PBD domain of GRP78 in the regulation of c-Src phosphorylation and found that c-Src phosphorylation was almost not detected in HepG2 cells transfected with delATPase, indicating GRP78 facilitated c-Src phosphorylation through its ATPase domain (Figure 6e-6f).

### Targeting of c-Src decreased the LSF/TS axis

**Figure 7:**
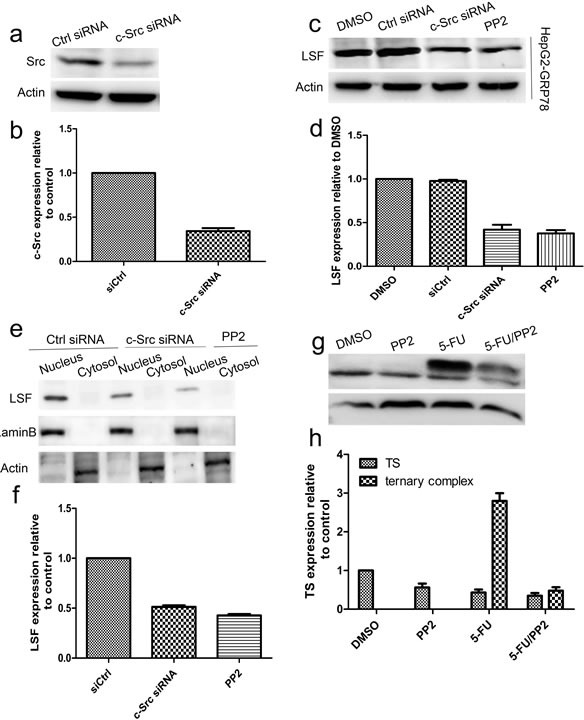
Targeting of c-Src decreased the LSF/TS axis **a.-b.** Western blot analysis of c-Src in HepG2-GRP78 cells transfected with siRNA against c-Src. **c.-d.** Western blot analysis of LSF in HepG2-GRP78 cells transfected with siRNA against c-Src or treated by PP2.**e.-f.** Western blot analysis of LSF in the cytosol and nuclear extracts in HepG2-GRP78 cells transfected with siRNA against c-Src or treated by PP2. **g.-h.** Western blot analysis of TS in HepG2-GRP78 cells treated with PP2 in the presence or absence of 5-FU.

It has been reported previously that c-Src plays critical roles in the resistance to many chemical agents in human cancers including pancreatic, colorectal, breast and liver cancer cells. For this reason, we examined whether c-Src regulates the expression of LSF. For this purpose, HepG2-GRP78 cells were transfected with siRNA against c-Src (Figure 7a-7b) and western blot analysis revealed that knockdown of c-Src caused a significant decrease in LSF level. This conclusion was further validated by the fact that inhibition of c-Src with PP2 (10μM) significantly decreased LSF expression in HepG2-GRP78 cells (Figure 7c-7d). Further analysis revealed that either transfection of c-Src siRNA or treatment with PP2 caused a significant decrease in LSF level in the nuclear extract as compared with that in control cells (Figure 7e-7f). Similar results were obtained by immunohistochemical staining in HepG2-GRP78 cells treated by c-Src siRNA or PP2 using anti-LSF antibody (Figure S3e). On the basis of these results, we next investigated whether c-Src upregulates the expression of TS using western blot and found that treatment of HepG2-GRP78 cells with PP2 significantly decreased TS expression as compared with that in cells treated with DMSO. Moreover, treatment with PP2 significantly decreased the level of the ternary complex of TS with FdUMP and CH2-THF (Figure 7g-7h).

### Inhibition of c-Src sensitizes HCC cells to 5-FU *in vitro* and *in vivo*

**Figure 8:**
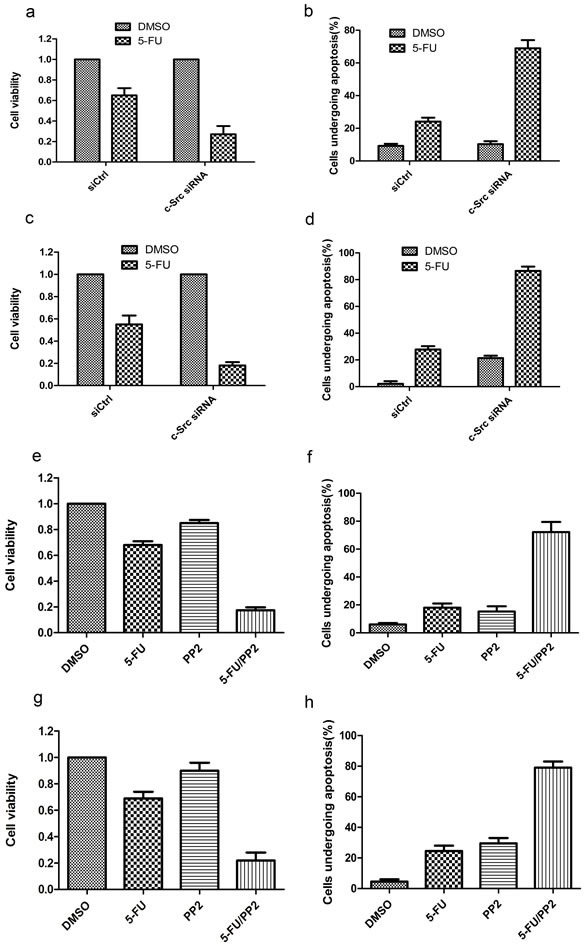
Targeting of c-Src sensitizes HCC cells to 5-FU *in vitro* **a.** Cell viability analysis of the sensitivity to 5-FU in HepG2-GRP78 cells transfected with c-Src siRNA. Data are representative of three independent experiments (mean±S.D.). **b.** Schematic show of flow cytometry analysis of cell apoptosis in HepG2-GRP78 cells transfected with c-Src siRNA when treated with 5-FU at a concentration of 10 μg/ml. Data are representative of three independent experiments (mean±S.D.). **c.** Cell viability analysis of the sensitivity to 5-FU in QGY-7703 cells transfected with c-Src siRNA. Data are representative of three independent experiments (mean±S.D.). **d.** Schematic show of flow cytometry analysis of cell apoptosis in QGY-7703 cells transfected with c-Src siRNA when treated with 5-FU at a concentration of 10 μg/ml. Data are representative of three independent experiments (mean±S.D.). **e.** Cell viability analysis of the sensitivity to 5-FU in HepG2-GRP78 cells treated with PP2 in the presence of 5-FU (10 μg/ml). Data are representative of three independent experiments (mean±S.D.). **f.** Schematic show of flow cytometry analysis of cell apoptosis in HepG2-GRP78 cells treated with PP2 in the presence of 5-FU (10 μg/ml). Data are representative of three independent experiments (mean±S.D.). **g.** Cell viability analysis of the sensitivity to 5-FU in QGY-7703 cells treated with PP2 in the presence of 5-FU (10 μg/ml). Data are representative of three independent experiments (mean±S.D.). **h.** Schematic show of flow cytometry analysis of cell apoptosis in QGY-7703 cells treated with PP2 in the presence of 5-FU (10 μg/ml). Data are representative of three independent experiments (mean±S.D.).

To investigate whether inhibition of c-Src sensitizes HCC cells to 5-FU, we knocked down c-Src expression in HepG2-GRP78 cells using siRNA against c-Src (Figure 7a-7b). Cell viability analysis showed that knockdown of c-Src significantly decreased the viability of HepG2-GRP78 cells when treated with 5-FU (Figure 8a). Flow cytometry analysis showed that treatment of HepG2-GRP78 cells transfected with c-Src siRNA with 5-FU significantly facilitated cell apoptosis as compared with control cells (Figure 8b, S4a). Similar results were obtained in QGY-7703 cells transfected with c-Src siRNA (Figure 8c-8d, S4c).

We next determined the effect of 5-FU in combination with PP2 on cell viability and apoptosis in HepG2-GRP78 cells and QGY-7703 cells. We found that treatment with 5-FU in combination with PP2 caused a significant decreased cell viability as compared with that treated with 5-FU or PP2 alone in HepG2-GRP78 (Figure 8e) and QGY-7703 cells (Figure 8g). Consistently, treatment with 5-FU in combination with PP2 caused a significant increased cell apoptosis as compared with that treated with 5-FU or PP2 alone in HepG2-GRP78 (Figure 8f, S4b) and QGY-7703 cells (Figure 8h, S4d).

**Figure 9:**
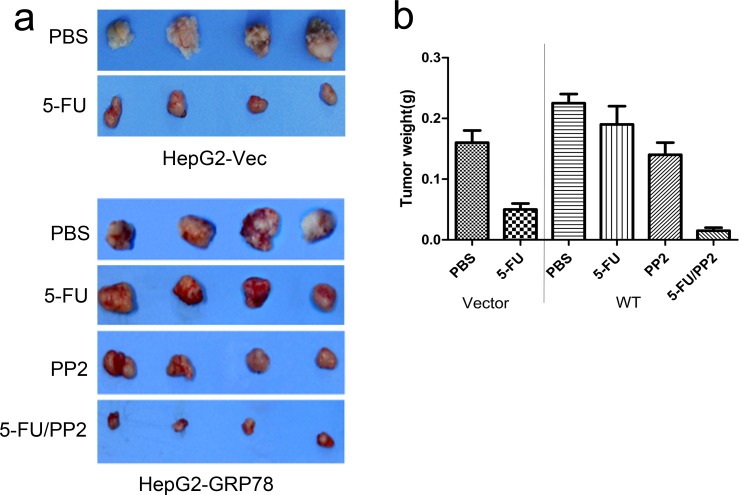
Targeting of c-Src sensitizes HCC cells to 5-FU *in vivo* **a.** *In vivo* analysis of the growth inhibitory effect of PP2/5-FU on HCC in an ectopic xenografts model. **b.** Diagram of tumor weight **g.** in an ectopic xenografts model when treated with PBS, 5-FU, PP2 or 5-FU in combination with PP2.

We next determined whether inhibition of c-Src could increase the sensitivity of HepG2 cells to 5-FU *in vivo* in an ectopic xenograft model. HepG2-GRP78 cells were injected subcutaneously into athymic nude mice. 2 weeks after injection, when tumor volume reached ∼100 mm^2^, mice were treated with intraperitoneal injections of PBS, PP2 (5mg/kg/d), 5-FU (50mg/kg/d) or PP2 /5-FU (5mg/kg/d, 50mg/kg/d) twice a week over 14 days. Consistent with *in vitro* experiments, 5-FU treatment caused a mild reduction in tumor weight in HepG2-GRP78 tumors relative to that in HepG2-Vec tumors. In contrast, treatment of HepG2-GRP78 tumors with 5-FU in combination with PP2 markedly reduced tumor weight (Figure 9a-9c).

## DISCUSSION

Acquired resistance to 5-FU is a common phenomenon and limits its clinical application in HCC. Although the mechanisms underlying this phenomenon have been the subject of intense investigation for many years and several mechanisms of acquired resistance to 5-FU have been proposed. Serval lines of evidence have established the critical role of the LSF/TS axis in the acquired resistance to 5-FU [17, 30, 31]. In this manuscript, we demonstrated that GRP78 could confer resistance to 5-FU by activating the c-Src/LSF/TS axis in HCC, identifying the critical role of GRP78 in the development of acquired resistance to 5-FU. Our results proposed a new explanation for the acquired resistance to 5-FU in HCC and may provide a method to overcome this phenomenon.

We have previously reported that GRP78 promoted the invasion and metastasis of HCC [32-34]. In addition, it maintains the viability of tumor cells and protects them from death under adverse condition. GRP78 is also involved in the resistance to chemotherapeutic drugs. Our present studies demonstrated that GRP78 could confer resistance to 5-FU in HCC. Cell viability analysis using HepG2 cells overexpressing GRP78, delATPase or delPBD indicated that overexpression of GRP78 or its delPBD resulted in a pronounced resistance to 5-FU; By contrast, HepG2 cells overexpressing delATPase significantly increased the sensitivity of HepG2 cells to 5-FU, demonstrating that GRP78 could cause the resistance to 5-FU through its ATPase domain in HCC.

Resistance to 5-FU could be raised through multiple mechanisms [35-37]. Many reports [30, 31]have demonstrated the central role of the LSF/TS axis in the development of 5-FU resistance. We found in this report that over-expression of GRP78 up-regulates the LSF/TS axis in HCC. Analysis of GRP78 and LSF expression in clinical samples has demonstrated a positive correlation between the expressions of these two proteins. In addition, the expression levels of GRP78 or LSF also showed a direct positive correlation to the stages and grades of HCC. In cellular level, over-expression of GRP78 up-regulates the expression of LSF and TS and promotes the translocation of LSF from cytosol into nucleus. However, knockdown of GRP78 down-regulates the expression of LSF and TS. Overexpression of WT-GRP78 or delPBD in HCC resulted in a significant increase in the expression of LSF and TS. Finally, the resistance to 5-FU conferred by GRP78 could be reverted by down-regulation of LSF using siRNA.

In an attempt tried to identify GRP78 interacting proteins. We found that GRP78 could interact directly with c-Src, documented by Y2H hybrid and GST pulldown assay. Indeed, the interaction between GRP78 and c-Src was confirmed with anti-EGFP co-immunoprecipitation and co-transfection of EGFP-tagged GRP78 and DsRed-tagged c-Src. Further analysis demonstrated that the interaction between GRP78 and c-Src is mediated by the ATPase domain of GRP78. Moreover, we found that GRP78 regulates the phosphorylation of c-Src through its ATPase domain. Collectively, these findings indicated that GRP78 could interact directly with c-Src and increase the activity of c-Src, this results were consistent with the works by other groups. It has been reported that c-Src plays critical roles in the development of resistance to many chemotherapeutic drugs including 5-FU and inhibition of c-Src could revert the resistance to 5-FU in pancreatic and colorectal cancers. We found that inhibition of c-Src using PP2 and siRNA down-regulates the expression of LSF and inhibited LSF translocation from cytosol into nucleus in HCC. These data suggested that c-Src is the mediator by which overexpression of GRP78 regulates the LSF/TS axis.

Finally, We found that inhibition of c-Src in combination with 5-FU treatment resulted in almost complete inhibition of cell growth both in HCC cells and a mouse ectopic xenograft model, demonstrating that c-Src plays a central role in the resistance to 5-FU conferred by GRP78.

In summary, we have identified c-Src as a GRP78 downstream protein, when activated, c-Src increased the expression of LSF and promoted the translocation of LSF from cytosol into nucleus, which in turn elevated the expression of TS, conferring the resistance to 5-FU. Our findings provided the fact that inhibition of c-Src could sensitized HCC cells to 5-FU, suggesting that it may be exploited as an effective adjuvant therapy for HCC.

## MATERIALS AND METHODS

### Cell culture

Human hepatocellular carcinoma cell lines QGY-7703, SMMC7721, PLC, Hep3B, HepG2 and Malhavu were purchased from the Type Culture Collection of the Chinese Academy of Sciences (Shanghai, China). All cell lines were cultured in RPMI-1640 medium supplemented with 10% FBS (Gibco, Life Technologies, USA) and antibiotic-antimycotic (Gibco, Life Technologies, USA).5-FU was obtained from Sigma-Aldrich (St. Louis, MO, USA). PP2 were the product of Selleck Chemicals (Houston, TX, USA).

### RNA interference

The siRNAs against GRP78 were synthesized by Genechem Corporation (Shanghai, China). The sequences of sense strands were as follow: GRP78 siRNA1: 5′-GGAGCGCAUUGAUACUAGAUU-3′; GRP78 siRNA2: 5′-GACGCUGGAACUAUUGCUUU-3′. siRNA against LSF and c-Src consisting of pools of three to five target-specific 19-25 nt was purchased from Santa Cruz. Transfections were performed in six-well plates following Lipofectamine2000's instructions. Cells were transfected with 4μg of siRNAs. 10μl Lipofectamine2000 were used for each well. The control siRNA was also purchased from Santa Cruz.

### Transfection

HepG2 cells stably overexpressing GRP78 or its mutant were generated by transfection of EGFP tagged WT-GRP78 or its ATPase or PBD domain deleted mutants. Transfection was performed according to lipofectamine2000's instruction. Briefly, Cells were cultured to 90% confluence in a six-well plates and transfected with 4 μg plasmid using 16 μl lipofectamine2000 (1:4 ratio). After 24h after transfection, the transfection efficiency was observed using inverted fluorescent microscope. Cells overexpressing GRP78 or its mutant were selected in complete medium containing 400μg/ml G418 for 2-3 weeks and identified by western blot using anti-GFP antibody. The plasmid used in this experiment were as follows: pEGFP-N1, pEGFP-N1-WT GRP78, pEGFP-N1-delATPase and pEGFP-N1-delPBD. For observation of the cellular localization of delATPase and delPBD mutants, HepG2 cells transfected with pEGFP-N1-WT GRP78, pEGFP-N1-delATPase and pEGFP-N1-delPBD were stained with TRITC-conjugated ER tracker after 48h of transfection and the cellular localization was observed with inverted microscope. For co-transfection, Confluent cells were transfected with 4 μg each recombinants. 20 μl lipofectamine2000 was used in each transfection reaction. The plasmids we used in co-transfection were: pEGFP-N1-WT GRP78, pEGFP-N1-delPBD, pEGFP-N1-delATPase and pDsRed-c-Src.

### Western blot

Preparation of whole cell lysates and western blot analysis was performed as described. The primary antibodies used were anti-GRP78(sc-1050), anti-actin(sc-1616), anti-LaminB(sc-6216)(1:1000, Santa Cruz Biotechnology, USA), anti-EGFP (1:1000, Origene Rockville, USA, TA150052), anti-LSF (1:500, Abcam, Cambridge, UK;ab180033), anti-TS(ab7398), anti-ERK(ab17942), anti-p-ERK(ab47339), anti-Akt(ab179463), anti-p-Akt(ab38449)(1:1000, Abcam, Cambridge, UK), anti-Gab1(#3232),anti-p-Gab1(#3233),anti-c-Src(#2123), anti-p-c-Src(#6943)((1:500, Cell signaling, Danver, USA). For analysis of the translocation of LSF from cytosol to nucleus, the nuclear extract and cytosol extract were isolated using NE-PER nuclear and cytoplasmic extraction reagents (Pierce, thermo fisher, France).

### Human tissue specimens

All the surgery resected tissue samples were obtained from the Department of Gastroenterology of the General Hospital of Chinese Liberation Army. All the studies related to these tissue samples were in compliance with Helsinki Declaration. The differentiation extents were re-evaluated by two pathologists. The clinicopathological data were summarized in Table 1. It is worth to note that none of these patients has received chemotherapy or irradiation before surgery.

### Immunohistochemistry

Immunohistochemistry was performed as previously reported. The dilution ratio of primary antibodies were 1:100. Antigen was retrieved by high pressure for 2 min in citrate buffer (0.01 M sodium citrate, pH 6.0). All sections were examined and scored independently by two investigators without any knowledge of the clinicopathological data of the patients, at least 5 fields were randomly chosen. The expression of Grp78 and LSF were evaluated according to the ratio of positive cells and staining intensity per field and scored as 0 for staining less than 5%, 1 for staining of 5 to 10%, 2 for staining of 10 to 50%, 3 for staining >50%. Intensity was graded as follows: 1, weak; and 2, strong staining. A total score of 0 to 6 was calculated and the scores were designated as 1(score: 0-1), 2 (2-4), and 3(5-6).

### Anti-EGFP immunoprecipitation

Cell lysates contain at least 1000 μg of protein from each sample were pre-cleared with 50 μl of protein A-Sepharose beads for 1 h at 4°C and incubated with 5 μg of anti-EGFP antibody (Abcam, Cambridge, UK) overnight at 4°C on a rotator. The total volume of this reaction was 1ml. Following antibody incubation, 100 μl of protein A sepharose beads (50% slurry) were added and rotated at 4°C for 3 h. The beads were then centrifuged at 12,000g for 5min and washed for 5 times with 1% NP40 lysis buffer. The precipitates were eluted by adding of 50 μl of 1× SDS-PAGE sample loading buffer (50 mm Tris-HCl, pH 6.8, 100 mm DTT, 2% SDS, 0.1% bromphenol blue, 10% glycerol), followed by boiling at 100°C for 5 min. The supernatant obtained after centrifugation was resolved by 10% SDS-PAGE and subjected to Western blot analysis.

### GST pulldown assay

Whole cell lysates were isolated as described previously. The clarified whole cell lysates were incubated with 50 μl of a 50 % slurry of glutathione-Sepharose 4B (GE Healthcare, USA) and 25 μg GST for 1 h at 4°C. Resins bounded with GST-GRP78, GST-delPBD,GST-delATPase or GST were incubated with whole cell lysates containing 1 mg protein extract overnight at 4°C on a rotator. The resins were then washed 5 times with ice-cold lysis buffer. Proteins were eluted by adding 25 μl of 2 × Laemmli sample buffer at 100°C for 5 min and centrifuged for 5 min at 12,000 g. The supernatant was resolved by SDS-PAGE and subjected to Western blot analysis using anti-c-Src (#2123, Cell signaling).

### RT-PCR

Conventional RT-PCR was performed as previously reported. The primers we used were as follows:

LSF: Forward 5′-AATTGCTCAGCTTTTCAGCA-3′; Reverse 5′-CCCTCTGTGTGTACCACAA-3′.

### Yeast two-hybrid screening

All vectors, yeast strains, reagents, and methods were derived from the MATCHMAKER Two-Hybrid System (Clontech, Palo Alto, CA, USA). PCR-generated chickGRP78 full length was inserted into pGBKT7 in-frame with the DNA binding domain of GAL4 at its 3′ end, to generate pGBKT7-GRP78. Screening was performed using the GRP78-Gal4 DNA binding domain fusion protein expressed from pGBKT7-GRP78 as the bait. A human Fetal Liver cDNA Library was constructed and screened according to the manual. Yeast AH109 was co-transformed with pGBKT7-GRP78, liver cancer library cDNA, and linearized pGADT7-Rec, using a high-efficiency lithium acetate/poly-ethylene glycol method. Positive colonies were selected on SD/-Ade/-His/-Leu/-Trp/plates and assayed for b-galactosidase activity. Secondary screens were performed in a similar manner to minimize false positives, and the bait was expelled through saturation growth in SD-Leu. Positive interacting colonies were recovered, sequenced, and matched to known sequences using BLAST (National Centre for Biotechnology Information, NCBI). To map the interacting domains on GRP78 or SRC, different truncated GRP78s and SRCs were inserted into pGBKT7 in-frame with the DNA binding domain of GAL4 at its 3′end and pGADT7-Rec respectively, then cotranfected into Yeast AH109 and positive interactions were selected on SD/-Ade/-His/-Leu/-Trp/plates and assayed for β-galactosidase activity.

### Cell viability assay

Cells were cultured at 5,000 cells per well in 96-well tissue culture plates. After 24 h after plating, cells were washed 3 times with PBS and then treated with the 5-FU for 72 hours in RPMI-1640 containing 0.5% FBS. At the end of the culture period, cells were washed with ice cold PBS, the MTT reagents were added according to the manufacturer's instructions and the absorbance was measured at 570 nm using a microplate reader. Mean values were calculated from three independent experiments. Cell viability is expressed as the ratio of the absorbance of cells treated with 5-FU and that of cells treated with DMSO. To investigate the chemosensitizing effect of c-Src kinase inhibitors, 5-FU was applied to cell culture following pretreatment with PP2 for 24 h, which was then removed and cells were treated for the subsequent 48 h with 5-FU alone.

### Transwell assay and wound healing assay

*In vitro* cell invasion and migration were analyzed using transwell assay and wound healing assay as previously described[32]. The experiments were repeated for 3 times and the data were represented as mean±S.D.

### Flow cytometry

Cells were seeded in complete medium in 6-well culture plates at a density of 10^6^ cells per well. After 24h of plating, cells were washed 3 times with PBS and then treated with the indicated drugs for 48 hours in RPMI-1640 containing 0.5% FBS. After 72 hours, cells were trypsinized, stained with Annexin V-FITC and propidium iodide for 30min, fixed with 70% ethanol and analyzed by flow cytometry (FACSCalibur^TM^, Becton Dickinson). The cells undergoing apoptosis were determined according to the manufacturer's instructions. Experiments were repeated for three times.

### Mice and *in vivo* tumor studies

All animal procedures were performed according to the national animal experimentation guidelines. Six-week-old female nude mice (BLAB/c-nude) were purchased from the institution of animal experimentation of Liaoning medical college. Cells(1×10^7^ ) were resuspended in 100μl PBS and injected subcutaneously into the dorsal flank of 24 mice(4 for each group). 2 weeks after injection, when tumor volume reached ∼100 mm^2^, mice were treated with intraperitoneal injections of PBS, PP2 (5mg/kg), 5-FU (50mg/kg) or PP2/5-FU (5mg/kg/50mg/kg) twice a week over 14 days. Subsequently, tumors were harvested and the size and weight of these tumors were evaluated.

### Statistical analysis

Comparison of the data was performed using one way ANOVA, student *t*-test and chi-square test. A *p*-value less than 0.05 was considered to be statistically significant.

## SUPPLEMENTARY MATERIAL FIGURES


