# Supplementary material for: GRP78 confers the resistance to 5-FU by activating the c-Src/LSF/TS Axis in hepatocellular carcinoma
Source: Oncotarget. 2015 Sep 10;6(32):33658–74. doi: 10.18632/oncotarget.5603 (PMC4741793; doi:10.18632/oncotarget.5603)
Supplement: Supplementary file 1 [file oncotarget-06-33658-s001.pdf]

GRP78 confers the resistance to 5-FU by activating the c-Src/LSF/TS Axis in hepatocellular carcinoma

Supplementary Material

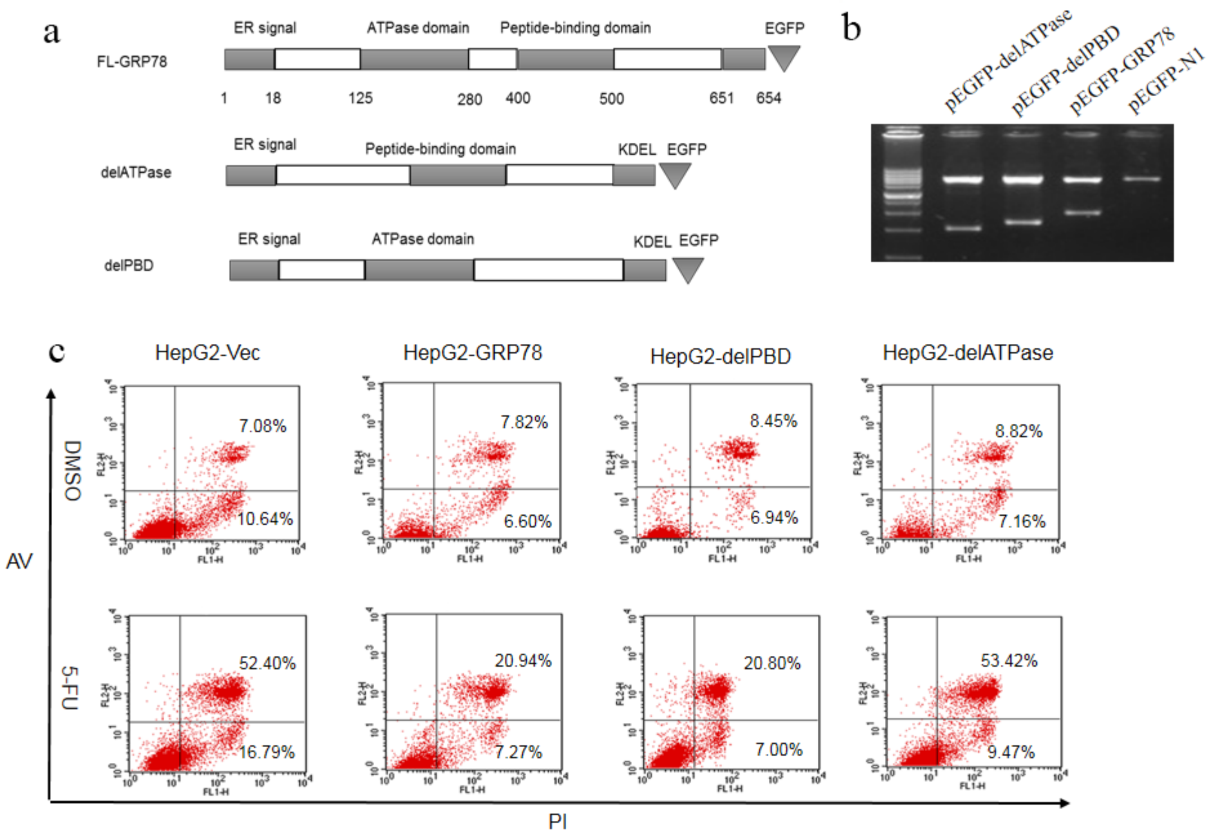

Fig.S1: (a) Schematic show of GRP78 and its ATPase or PBD domain deleted mutant. **(b)** Construction and identification of GRP78 and its ATPase and PBD deleted mutants. ATPase or PBD domain deleted mutants were constructed on the basis of pEGFP-N1-GRP78, verified by endonuclease cleavage using EcoRI and HindIII and separated by 1% agarose electrophoresis. **(c)** Flow cytometry analysis of the sensitivity to 5-FU in HepG2 cells transfected with EGFP-tagged GRP78, delPBD or delATPase. These experiments were repeated for three times.

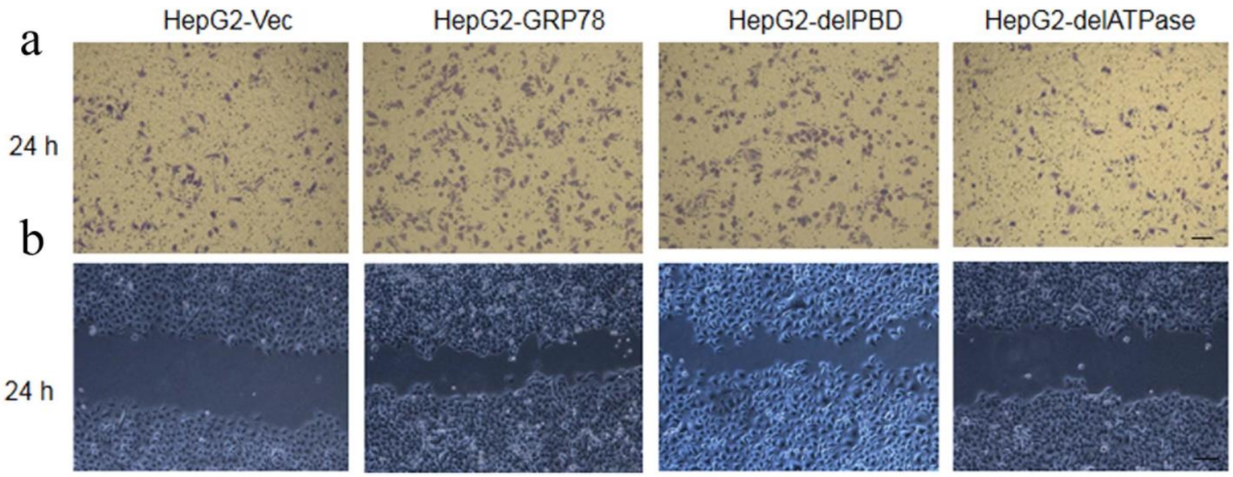

Fig.S2: (a-b) GRP78 promotes the invasion and migration of HepG2 cells through its ATPase domain. **(a)** Transwell analysis of the invasion of HepG2 cells transfected with EGFP tagged GRP78, delPBD or delATPase (Scale bar: 50 μM). **(b)** Wound healing analysis of the migration of HepG2 cells overexpressing EGFP tagged GRP78, delPBD or delATPase (Scale bar: 50 μM).

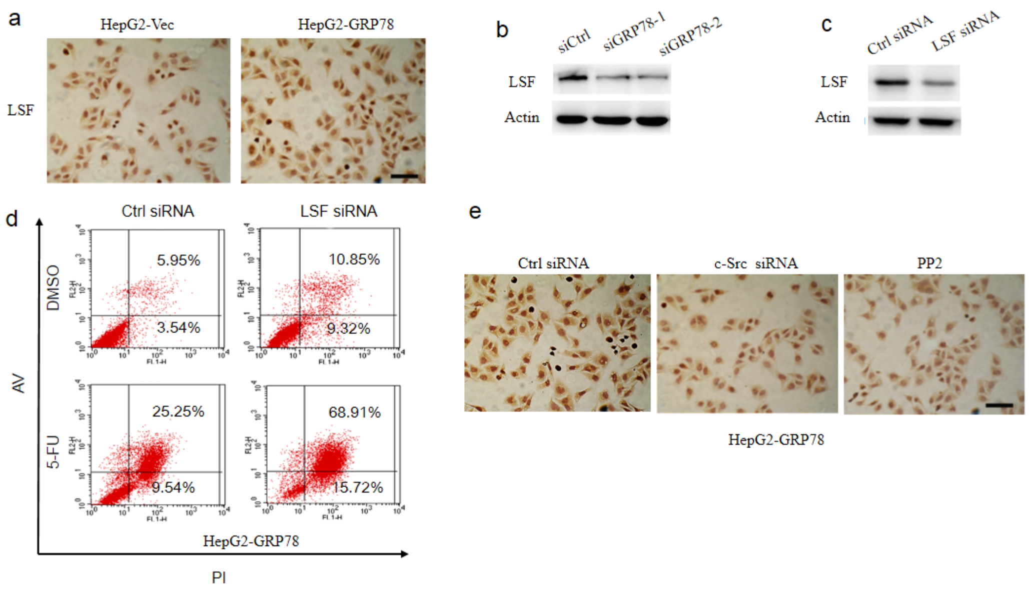

Fig.S3: (a) Immunocytochemical staining of LSF in HepG2-GRP78 or HepG2-Vec cells. (b) Western blot analysis of LSF in QGY-7703 cells transfected with siGRP78(Scale bar: 50 μM). (c) Western blot analysis of LSF level in HepG2-GRP78 cells transfected with siRNA against LSF.(d) Flow cytometry analysis of apoptosis induced by 5-FU in HepG2-GRP78 cells transfected with siRNA against LSF.(e) Immunocytochemical staining of LSF in HepG2-GRP78 cells treated with siRNA against c-Src or PP2(Scale bar:50 μM).

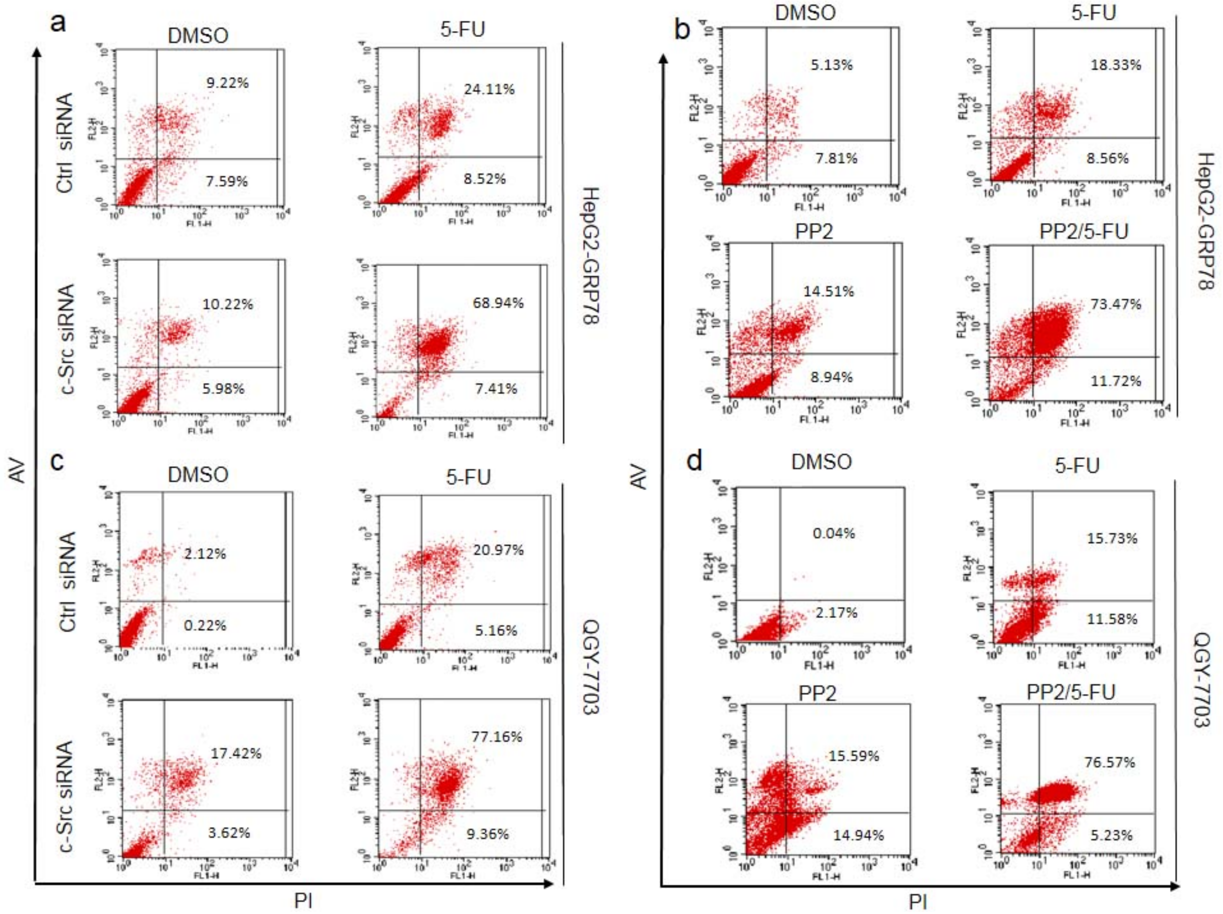

Fig.S4: (a) Flow cytometry analysis of apoptosis induced by 5-FU in HepG2-GRP78 cells transfected with siRNA against c-Src. (b) Flow cytometry analysis of apoptosis induced by 5-FU in HepG2-GRP78 cells treated with 5-FU, PP2 or 5-FU in combination of PP2. (c) Flow cytometry analysis of apoptosis induced by 5-FU in QGY-7703 cells transfected with siRNA against c-Src. (d) Flow cytometry analysis of apoptosis induced by 5-FU in QGY-7703 cells treated with 5-FU, PP2 or 5-FU in combination of PP2.

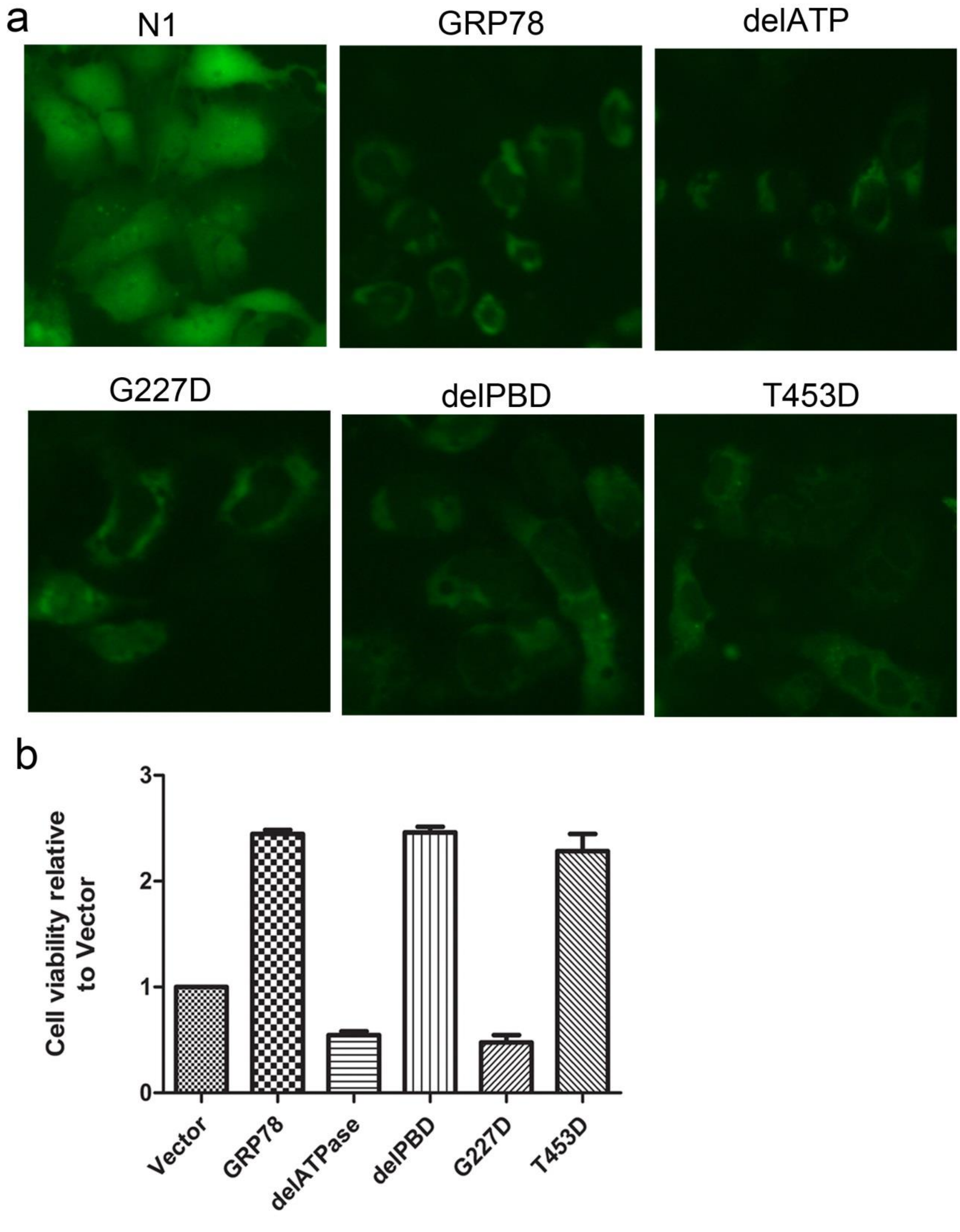

Fig.S5: (a) cellular localization of G227D, T473D, delATP and delPBD in hepG2 cells. (b). Cell viability analysis of hepG2 cells overexpressing G227D, T473D, delATP and delPBD.
